# Metabolomic Analysis Reveals Insights into Deterioration of Rice Quality during Storage

**DOI:** 10.3390/foods11121729

**Published:** 2022-06-13

**Authors:** Qian Wang, Dong Zhang, Luyao Zhao, Jianlei Liu, Bo Shang, Weiqiao Yang, Xiaoliang Duan, Hui Sun

**Affiliations:** Academy of National Food and Strategic Reserves Administration, Beijing 100037, China; wangq@ags.ac.cn (Q.W.); zd@ags.ac.cn (D.Z.); zly@ags.ac.cn (L.Z.); ljl@ags.ac.cn (J.L.); shb@ags.ac.cn (B.S.); ywq@ags.ac.cn (W.Y.); dxl@ags.ac.cn (X.D.)

**Keywords:** rice, storage, metabolomic, quality, OPLS-DA

## Abstract

To determine the changes in the quality of rice during storage, this study investigated the comprehensive metabolomic profiles of Nanjing 9108 (typical *japonica* rice) and Jianzhen 2 (typical *indica* rice) varieties in China, using metabolomics. A total of 13 categories of 593 metabolites including lipids (134 species), phenolic acids (78 species), flavonoids (70 species), alkaloids (67 species), organic acids (64 species), amino acids and derivatives (64 species), saccharides and alcohols (44 species), nucleotides and derivatives (37 species), vitamins (14 species), lignans and coumarins (9 species), tannins (2 species), terpenoids (2 species), and others (8 species) were identified in both varieties. The result showed significant changes in 204 metabolites in Nanjing 9108, while only 26 were altered in Jianzhen 2 during storage. These metabolites involved 46 metabolic pathways. The TCA cycle, linoleic, and α-linolenic acid metabolic pathways were unique in Nanjing 9108. Finally, the results of quantitative mass spectrometry of 11 metabolites provided insight into biomarkers associated with quality deterioration of rice. This study provides insights into the mechanism of deterioration in the quality of rice during storage.

## 1. Introduction

Rice (*Oryza sativa* L.) is a native and fast-growing crop cultivated in Asian countries with a high content of carbohydrates and proteins. It is consumed as a staple food by people living in this region. Factors such as crop variety, climatic conditions, pre-harvest operations, and technical management affect grain quality. However, postharvest practices play an equally essential role [1]. As an important postharvest process, a long or short time after harvest is necessary to maintain a continuous market supply of rice. However, storage generally causes changes in nutrients, physical properties, and chemical composition, consequently leading to deterioration in the cooking and eating quality of rice [2,3]. The chemical composition of rice determines the taste and nutritional value of rice to a great extent and is an important basis for the evaluation of rice quality [4].

Carbohydrates (starch), protein, and lipids are closely related to the quality of rice [5,6,7]. Currently, the factors affecting the quality of rice are mainly attributed to disordered cell membranes, protein deformations, or a loss of activity, leading to the production of excessive reactive oxygen species (ROS) due to abiotic stress, which results in an oxidative imbalance in rice [8,9,10]. Besides this, rice consumption involves many solutes (sugar and alcohol) to maintain the balance. These substances play an essential role in stabilizing protein and cell structure or maintaining cell expansion through infiltration, which directly affects the viscosity of rice after cooking [11]. Nonetheless, the cell membrane fluidity is altered by changing the cell membrane components (such as the increase of free fatty acids) to restore balance. These fatty acids are degraded into low-molecular-weight substances such as free fatty acids, aldehydes, ketones, and acids with an unpleasant odor, resulting in a reduced eating quality of the rice [12,13]. We explored the changes in non-starch and starch lipids of two rice varieties with high eating quality during storage based on untargeted lipidomic approaches and found that non-starch lipids (including glycerolipids (TG), glycerophospholipids, and saccharolipids) and starch lipids exhibited contrasting changes in Nanjing 9108 and Jianzhen 2 [14]. Metabolomics analysis is used to investigate the end products of gene expression with low molecular weight (<1000 Da) in living organisms [15], which reveals comprehensive changes in metabolites. This approach is generally used for the characterization of rice aging and traceability to provide a potential pathway and determine the changes in biological phenotypes [10,16,17]. However, the analysis of metabolites profiles of rice belonging to different varieties during storage has yet to be reported.

Nanjing 9108 (*japonica* rice) and Jianzhen 2 (*indica* rice), two varieties with large planting areas in Jiangsu and Hubei province, are popular rice and excellent edible varieties in China. In the current study, a non-targeted metabolomics method was employed to investigate the alterations in metabolites profiles between freshly harvested and stored rice using UPLC-ESI-MS/MS. Then, the identified metabolites and metabolic pathways were fully analyzed by MetaboAnalyst 5.0 software. Individual levels of d-sucrose, d-fructose, d-glucose, d-trehalose, succinic acid, fumaric acid, citric acid, l-glycine, glutathione (reduced form, also named as GSH), l-cysteine, 13-KODE, and 9(S)-HOTrE were qualified via MS/M. Finally, the changes in metabolite profile and the differences and relationship between the deterioration of the quality of the two varieties were analyzed. This analysis complements our previous report on rice lipidomics [13,14] and contributes to a better understanding of the role of different metabolites and biomarkers in rice quality deterioration.

## 2. Materials and Methods

### 2.1. Reagents, Materials, and Storage Conditions

Methanol, acetonitrile, ultrapure water, acetone, and formic acid (LC-MS grade) were obtained from Thermo Fisher Co., Ltd. (Shanghai, China). Reference *japonica* and *indica* rice were provided by our research team at the Academy of National Food and Strategic Reserves Administration (Beijing, China). Other reagents with chemical grades were obtained from Beijing Chemical Reagents Company (Beijing, China).

Paddy *japonica* rice (Nanjing 9108) and *indica* rice (Jianzhen 2) were collected from local farms in the Jiangsu and Hubei provinces of China in October of 2018. After harvest, 50 kg of each rice variety (50 kg) was air-dried in a dark open field until the moisture content was 13% approximately, and then packed and sent to the laboratory (Beijing, China) promptly. Paddy rice was packed randomly in 3 aluminum foil bags to avoid light and pest infection after delivering to the laboratory in Beijing. The packed rice samples were stored in a climate-controlled chamber (100 × 100 × 100 cm) under atmospheric pressure from October 2018 to March 2020 (540 days). Average temperature and relative humidity during storage were 20 °C and 27%, respectively (Figure S1). The rice samples were collected at the beginning of storage and 540 days later. The samples were stored at −80 °C before analysis. Nanjing 9108 and Jianzhen 2 are referred to as NJ and JZ, and the fresh and stored groups are expressed using F and S, respectively. A comparison between NJF vs. NJS and JZF vs. JZS was performed in the following experiments. The fatty acid levels of NJF and JZF were 15.4 and 16.4 mg/100 g (dry base), respectively. After 540 days of storage, fatty acid levels of NJ and JZ were 37.0 and 17.3 mg/100 g (dry base). According to the “guidelines for evaluation of paddy storage charter” (GB/T 20569-2006), the fatty acid value of JZ indicated good storage quality, while NJ showed moderate storage quality, with a tendency to decline.

### 2.2. Analysis of Eating Quality, Texture, and RVA

A rice taste meter was used to evaluate the eating quality of rice with slight modifications according to Xu, et al. [18]. Rice samples were dehulled with a JDMZ-100 rice huller (Beijing Dongfu Jiuheng Instrument Technology Co., Ltd., Beijing, China) and milled to 90% with a CBS300 rice miller (SATAKE, Kawagishi, Japan). Milled rice (400 g) was washed three times with distilled water and kept at room temperature for 30 min for soaking before cooking (rice: water = 1:1.4). The cooked rice mixture was obtained after boiling for 35 min and warm holding for 15 min.

The eating quality (appearance, taste, and comprehensive scores) of milled rice were determined using an STA1B device (Satake Corp., Hiroshima, Japan). The detailed procedure was described by Chen, et al. [19]. Texture characteristics (hardness, stickiness, balance degree, and elasticity) were determined using the RHS1A device (Satake Corp., Hiroshima, Japan). Each sample was analyzed three times in parallel.

A rapid viscosity analyzer was used to determine the pasting properties of rice flour during storage. RVA was conducted to determine the pasting properties of rice flour using a Super 4 RVA (Newport Scientific, Warriewood, Australia). A detailed procedure was performed following the method of Zhu, et al. [20]. Briefly, 3.0 g of rice flour (ground after dehulling) was weighed into aluminum RVA sample canisters and mixed with 25 mL of distilled water. The sample weight and water volumes were adjusted adequately according to the method. A 12.5 min analytical procedure was performed as follows: the sample was maintained at 50 °C (1 min), increased to 95 °C in 3.8 min, held for 2.5 min, cooled down to 50 °C in 3.8 min, and left for 1.4 min. The initial stirring speed was set at 960 r/min for 1 min, then decreased to 160 r/min for the rest of the program. Peak viscosity, trough viscosity, final viscosity, peak time, and pasting temperature were recorded by Thermo Cycle software. Breakdown and setback were calculated using Microsoft Excel software. Viscosity was reported as cP.

### 2.3. Metabolites Extraction

Metabolite extraction was carried out according to the procedure published previously with minor modifications [21]. First, 0.6 mL of 70% methanol was added into a test tube with 50 mg of rice flour (ground in liquid nitrogen) and vortexed every 30 min for 30 s, six times in total. Then, the extracted samples were placed in the refrigerator at 4 °C overnight. The supernatant was collected after centrifugation (12,000 rpm, 10 min) and filtered with organic PTFE membrane (0.22 μm) for UPLC-MS/MS analysis.

### 2.4. Non-Targeted Metabolomic Analysis

Metabolite detection was performed on a UPLC-ESI-MS/MS system (UPLC: Nexera X2, SHIMADZU, Tokyo, Japan; MS: Sciex Qtrap 4500, Applied Biosystems, CA, USA). The SB-C18 (1.8 µm, 2.1 × 100 mm) and a mobile phase (**A**: pure water with 0.1% formic acid; **B**: acetonitrile with 0.1% formic acid) at the flow rate of 0.35 mL/min was used to separate metabolites extracted from both rice varieties. The gradient elution program was as follows: 0–9 min, 5–95% B; 9–10 min, 95% B; 10.1–11 min, 95–5% B; 11–14 min, 5% B. The column oven temperature and injection volume were set to 40 °C and 4 μL, respectively.

Mass spectrometry was conducted in negative and positive ionization mode with the following conditions: ion source of turbo spray, source temperature of 550 °C, and the ion spray voltage of 5500 V (positive ionization mode)/−4500 V (negative ionization mode), ion source gas I, gas II, and curtain gas were set at 50, 60, and 25 psi, respectively. The mass accuracy was calibrated before the experiment using 3-Chloroaniline as the internal standard. The quality control (QC) analysis of samples (mixed all test samples with the same volume in triplicate) was used to determine the reliability of the method.

### 2.5. Targeted Metabolomic Analysis by LC-MS/MS

d-sucrose, d-fructose, d-glucose, d-trehalose, succinic acid, fumaric acid, citric acid, l-glycine, GSH, l-cysteine, 13-KODE, and 9(S)-HOTrE in both samples during storage were quantitatively evaluated using internal calibration. The detailed conditions were similar to those in Section 2.5. l-2-Chlorophenylalanine was adopted as the internal standard to assess the stability of the method. Three replicates were prepared for each group.

### 2.6. Statistical Analysis

Data analysis and metabolite identification were performed using the Metware database (Metware, Wuhan, China). The differential analysis was completed by *t*-test. *p* < 0.05 indicated statistically significant difference, while *p* < 0.01 represented extremely significant difference. Mass errors of MS and MS/MS were both less than 20 ppm. Significantly regulated metabolites between freshly harvested and stored rice were determined at *p* < 0.05, FC > 2, and VIP scores > 1.0. OPLS-DA was conducted using MetaboAnalyst 5.0 software, while permutation tests (*n* = 200) of the OPLS-DA model were performed using the Metware platform (https://cloud.metware.cn/, accessed on 18 December 2021). Besides this, the identified metabolites were annotated using the KEGG pathway database (http://www.kegg.jp/kegg/compound/, accessed on 27 December 2021), and the annotated metabolites were then mapped to the KEGG database for pathway analysis (http://www.kegg.jp/kegg/pathway.html, accessed on 27 December 2021). The detailed data treatment processing was described in Supplemental material S2.

## 3. Results

### 3.1. Characteristics of Eating Quality, Texture, and RVA of the Two Rice Varieties

**Figure 1:**
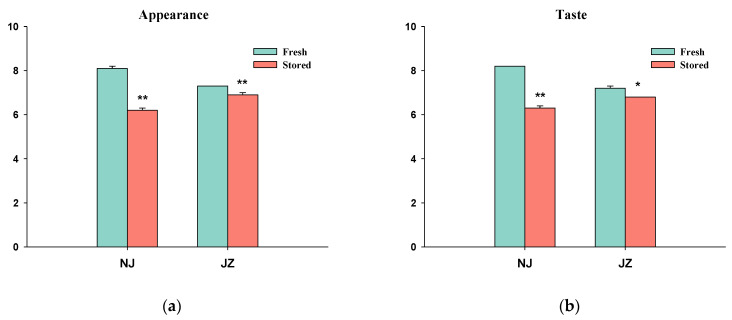

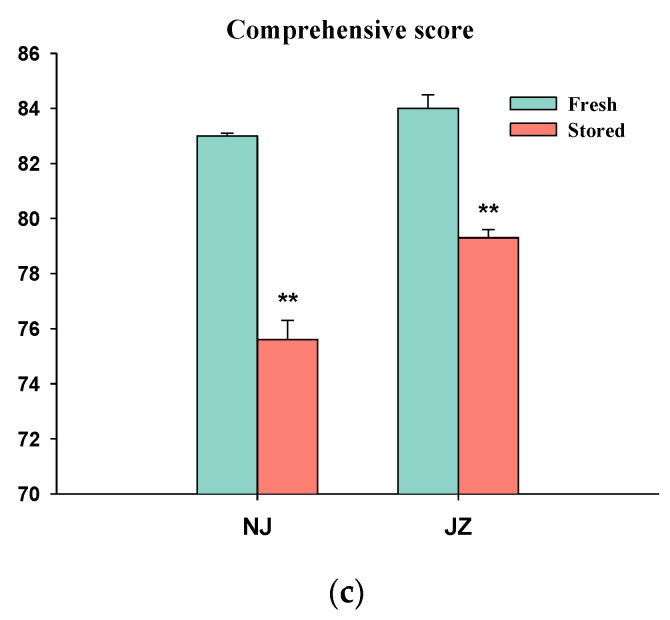
The eating quality of rice samples in this study. (**a**) Appearance; (**b**) Taste; (**c**) Comprehensive score. (*n* = 3, *: means the stored group is significantly different compared with the fresh group in the same variety when α = 0.05; **: means the stored group is significantly different compared with the fresh group in the same variety when α = 0.01).

Eating quality exported by STA1B, including appearance, taste, and comprehensive score is generally used to determine the eating quality of rice. As illustrated in Figure 1, the scores of these three indices decreased with storage in both varieties. The comprehensive scores of these two varieties decreased after 540 days of storage with a reduction rate of 8.9% and 6.8%, respectively. Figure S2 shows the skin color of unhulled rice and brown rice evenly distributed in a fresh sample. After 540 days of storage, the color of glume and brown rice of the two varieties turned yellow. The endosperm of some brown rice in NJS appeared milky white, and some JZS rice grains were close to dark brown. 

To gain insight into the eating quality difference between stored and fresh rice samples, the texture parameters representing the hardness and viscosity of rice were determined. Data in Table 1 show that the stored rice had a significantly higher hardness and stickiness when compared with that of fresh rice in both cultivars. Starch gelatinization is an important factor affecting rice taste during cooking, and RVA is a group of indicators designed to measure the gelatinization characteristics of starch (Table 1). The peak, trough, and final viscosity significantly increased during storage, as well as the breakdown and setback of two varieties. After storage, there was no significant difference in peak time and pasting temperature of NJ. However, a slight decrease in peak time and an increase in pasting temperature were observed during the storage of JZ.

### 3.2. Overview of Metabolic Profiles

A non-targeted metabolomic approach based on UPLC-MS/MS in both positive and negative ionization modes was used to determine the metabolite profiles of freshly harvested and stored rice. The QC analysis of both rice varieties was determined by identifying a total of 638 metabolites, including 347 and 290 features in positive and negative ionization modes, respectively. Relative standard deviations (RSDs) of peak area above 25% filtered in all metabolic ions were used to ensure the accuracy of the analysis. Finally, 13 categories of 593 metabolites including lipids (134 species), phenolic acids (78 species), flavonoids (70 species), alkaloids (67 species), organic acids (64 species), amino acids and derivatives (64 species), saccharides and alcohols (44 species), nucleotides and derivatives (37 species), vitamin (14 species), lignans and coumarins (9 species), tannins (2 species), terpenoids (2 species) and others (8 species), were identified in both varieties. Table S1 lists all the measured metabolites filtered in this study.

**Figure 2:**
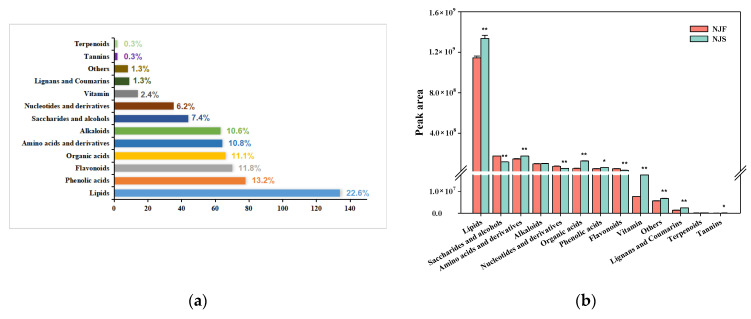

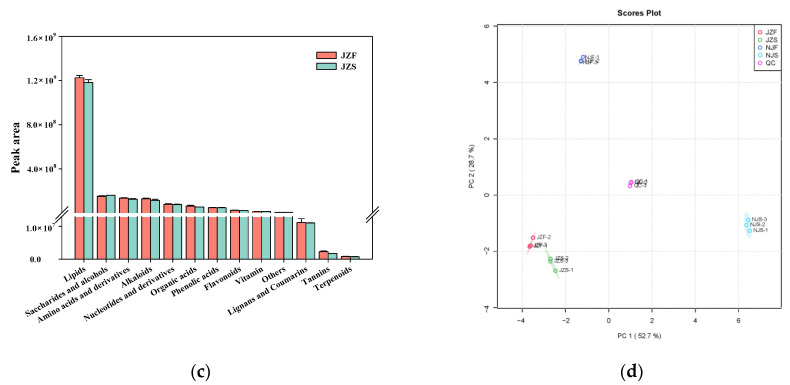
Overview of metabolic profiles. (**a**) Metabolite categories detected in the samples; (**b**) The analyzed peak area comparison in NJ during storage; (**c**) The analyzed peak area comparison in JZ during storage; (**d**) Principal component analysis (PCA) of metabolic profiles in all groups. (*n* = 3, *: means the stored group is significantly different compared with the fresh group in the same variety when α = 0.05; **: means the stored group is significantly different compared with the fresh group in the same variety when α = 0.01).

Mapping the total 593 metabolites to KEGG pathways revealed that lipids constituted the maximum percentage of the individual category (22.6%), while tannins (0.3%) and terpenoids (0.3%) were negligible (Figure 2a). Among them, 364 metabolites had KEGG ID information after matching the KEGG database. The relative abundances of metabolites in NJ and JZ are shown in Figure 2b,c. Most of the metabolites in Table S1 were involved in primary metabolism. A few secondary metabolites reflected the nutritional value, quality, and physiological state of grains during storage.

Unsupervised PCA analysis was used to evaluate the differences in the metabolic spectrum of 593 metabolites among different samples. According to the PCA results, the four rice samples fall into four groups clearly with PC1 (52.7%) and PC2 (28.7%). This demonstrated that the presence of marked variances in metabolites among groups depends on phenotype and species. Especially, separation was more obvious in NJS vs. NJF group than in JZS vs. JZF group (Figure 2d).

In addition, the heatmap revealed the changes in metabolites of the four samples, and Figure S3 shows the significant difference in metabolite abundance in all samples, which is consistent with the peak abundance difference observed in Figure 2b,c. Compared with JZ, the abundance of most metabolites in NJS was higher than in NJF. Following adaptation of the storage environment, the separation of metabolic levels indicates that different rice varieties may have experienced different metabolic processes.

### 3.3. Differences in Metabolic Profiles between Fresh Rice and Stored Rice

**Figure 3:**
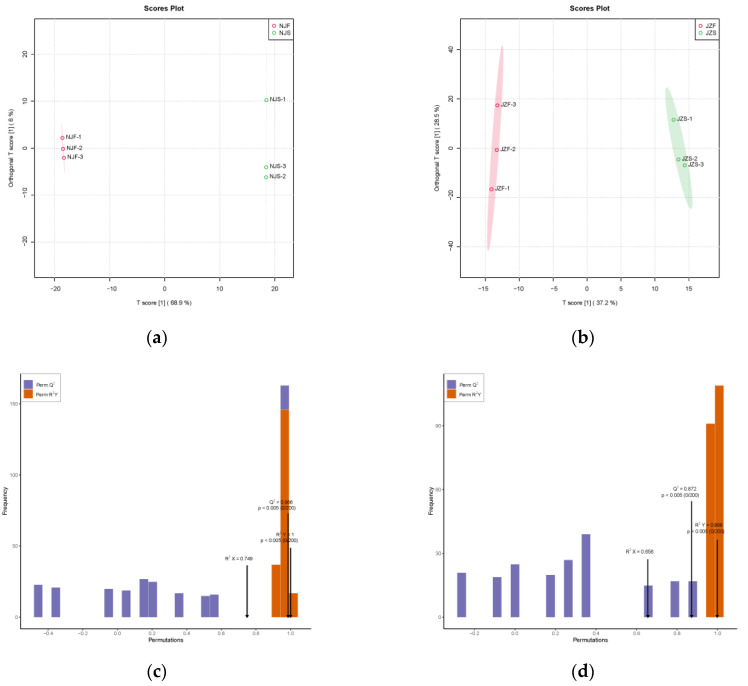
The metabolic variation between fresh and stored samples. (**a**) OPLS-DA score plot of different metabolites in NJS vs. NJF group. (**b**) OPLS-DA score plot of different metabolites in the JZS vs. JZF group. (**c**) Permutation testing of OPLS-DA in NJS vs. NJF group. (**d**) Permutation testing of OPLS-DA in JZS vs. JZF group.

The categories of metabolites in rice are listed in Table S1. To identify differentially expressed metabolites between freshly harvested and stored rice, the individual metabolites of two rice varieties from different storage times were compared using supervised OPLS-DA. As illustrated in Figure 3a,b, the abscissa represents the predicted principal component, while the ordinate denotes the orthogonal principal component. The interpretation rates of the predicted principal components of the NJS vs. NJF group and JZS vs. JZF group were 68.9% and 37.2% respectively, which indicated that the metabolites from the stored group were remarkably different from those of the freshly harvested rice. The interpretation rates of the orthogonal principal component of NJS vs. NJF group and JZS vs. JZF group were 6% and 28.5%, respectively. Therefore, based on OPLS-DA analysis, the metabolic characteristics vary among different rice varieties. The permutation test was conducted to further validate the model. The high values of R^2^X, R^2^Y, and Q^2^ in Figure 3c,d indicate the robustness of the OPLS-DA model for classification of freshly harvested and stored rice.

**Figure 4:**
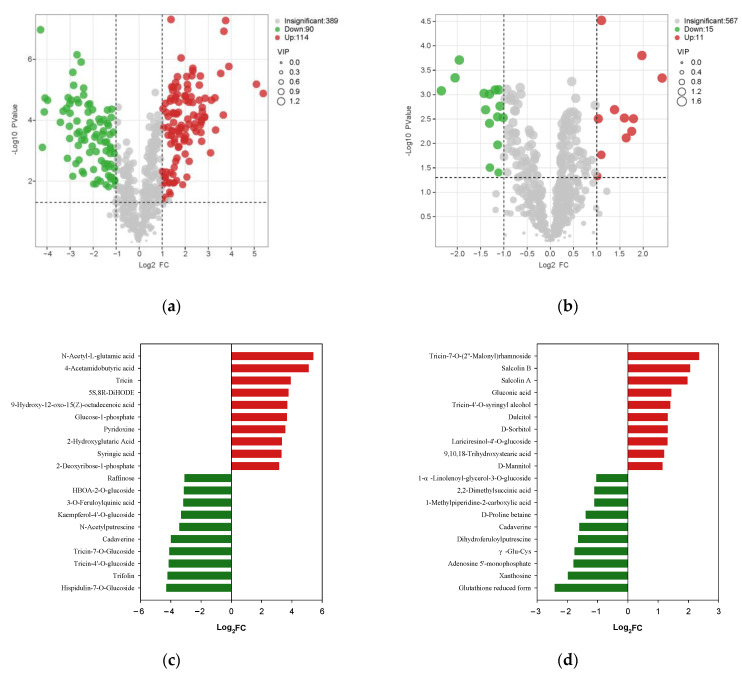
The metabolic variation between stored rice compared with fresh rice. (**a**) Volcano plot of the different metabolites in NJS vs. NJF group. (**b**) Volcano plot of the different metabolites in JZS vs. JZF group. (**c**) Up-regulation/down-regulation of the top 10 differential metabolites in NJS vs. NJF group. (**d**) Up-regulation/down-regulation of the top 10 differential metabolites in the JZS vs. JZF group.

Figure 4a,b visualize the differences in the expression of metabolites in samples before and after storage, as well as the statistical significance of the difference (α = 0.05). The red or green bubbles in Figure 4a,b represent metabolites with VIP > 1.0, which is considered relevant for group discrimination. Finally, 204 metabolites in NJS vs. NJF and 26 metabolites in JZS vs. JZF were considered to differ significantly (Table S2a,b, *p* < 0.05).

Among 204 differential metabolites in NJ, 114 were up-regulated and 90 were down-regulated (Figure 4a, Table S2a). The category of “phenolic acids” was the most altered metabolite in NJ (followed by flavonoids, saccharides and alcohols, organic acids, alkaloids, and lipids. The JZ variety included 15 up-regulated and 11 down-regulated metabolites (Figure 4b, Table S2b). Amino acids and derivatives were the most altered metabolites in JZ, followed by flavonoids and others (saccharides and alcohols). Among these metabolites, only 13 metabolites, including saccharides and alcohols (4 species), amino acids and derivatives (2 species), organic acids (2 species), nucleotides and derivatives (2 species), lipids (1 species), alkaloids (1 species) and tannins (1 species), changed similarly during storage in the two species (Table 2). Intriguingly, the variation of all these metabolites in NJ was more significant than in JZ. The top 20 metabolites of two varieties with the most remarkable change in FC fold after storage are shown in Figure 4c,d. *N*-acetyl-l-glutamic acid and hispidulin-7-*O*-glucoside were the most significantly up-regulated and down-regulated metabolites after storage for NJS vs. NJF, with FC fold of 41.949 and 0.051, respectively. Tricin-7-*O*-(2″-Malonyl)rhamnoside and GSH form were detected as the most significantly up-regulated and down-regulated metabolites after storage for JZS vs. JZF, with FC folds of 5.081 and 0.189, respectively.

### 3.4. Pathway Analysis of Differential Metabolites between Fresh and Stored Rice

**Figure 5:**
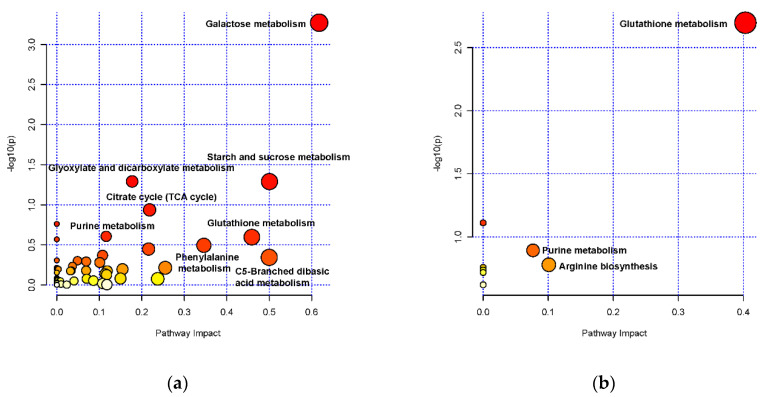
(**a**) KEGG pathway impact analysis showing altered metabolism in NJF vs. NJS group. (**b**) KEGG pathway impact analysis showing altered metabolism in JZF vs. JZS. The X-axis indicates pathway impact, and the Y-axis indicates enrichment. The dots with larger size and darker color represent the main pathway enrichment and higher pathway impact values, individually.

The pathway analysis of significantly altered metabolites was accomplished using *Oryza sativa japonica* as the pathway library and the results of Metabolite Set Enrichment Analysis results are listed in Table S3 and Figure 5. The pathway impact factors of all differential metabolites were 0.1–0.6 in both varieties and the up-regulated or down-regulated metabolites are listed in Table S2. These pathways are mostly associated with amino acid and carbohydrate metabolism (Table 2). In JZS vs. JZF, the metabolite differences enriched amino acid metabolic pathways, mainly including glutathione metabolism and arginine biosynthesis (Figure 5b). Higher levels of glutathione metabolism (e.g., GSH and cadaverine) in stored rice than in fresh rice were observed in the two species. The level of l-glycine was increased in NJS vs. NJF, while oxidized glutathione and γ-glutamylcysteine decreased in NJ and JZ, respectively. Another overlapping pathway of the two comparative groups was purine metabolism, in which adenosine monophosphate and xanthosine were significantly down-regulated in both varieties. Further, specific changes were observed in NJS vs. NJF (Figure 5a), such as metabolism of galactose, starch, and sucrose, carbon fixation in photosynthetic organisms, citrate cycle (TCA cycle), glycolysis/gluconeogenesis, and glyoxylate and dicarboxylate metabolism involving carbohydrates (stachyose, d-sorbitol, galactinol, d-sucrose, raffinose, dulcitol, d-mannose, d-galactose, d-glucose, d-trehalose, arbutin, glucose-1-phosphate, d-glucose, 3-phospho- d-glyceric acid, succinic acid, fumaric acid, L-malic acid, and citric acid), glutathione metabolism, arginine biosynthesis for amino acid metabolism (oxidized glutathione, l-glycine, and *N*-Acetyl-l-glutamic acid), linoleic acid metabolism, and α-linolenic acid metabolism for fatty acid metabolism (13(S)-HPODE, 12(13)-EpOME, 5S,8R-DiHODE, 7S,8S-DiHODE, 9-OxoODE, 13-KODE, 9,10-Dihydroxy-12,13-epoxyoctadecanoate, 9(S)-HOTrE, 13(S)-HOTrE, 9-Hydroxy-12-oxo-15(Z)-octadecenoic acid, and 12-OPDA). Pyrimidine metabolism related to phenylpyruvic acid and 2-hydroxyphenylacetic acid was also affected in NJ, which indicated the differential response mechanism of the two varieties under the storage environment.

### 3.5. Targeted Metabolomics Analysis by LC-MS/MS

**Figure 6:**
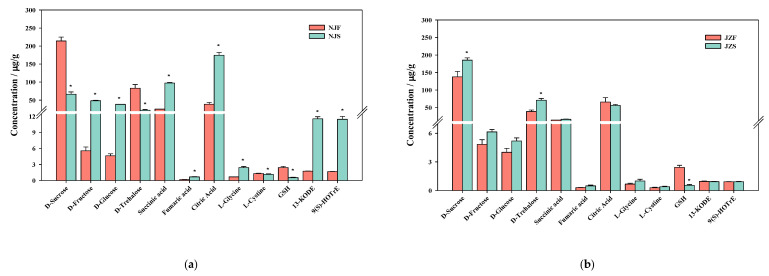
(**a**) Relative quantification of metabolites in NJF vs. NJS group. (**b**) Relative quantification of metabolites in JZF vs. JZS. *: means the stored group is significantly different compared with the fresh group in the same variety when α = 0.05.

In order to confirm the changes in metabolic pathways and potential biomarkers during storage, 11 metabolic compounds related to carbohydrates, amino acids, and fatty acid metabolism were quantified relatively (Figure 6). For the biomarkers mentioned in Section 3.4, seven carbohydrates, namely d-sucrose, d-fructose, d-glucose, d-trehalose, succinic acid, fumaric acid, and citric acid were determined. The level of d-trehalose was significantly decreased in NJ but significantly increased in JZ. Succinic acid, fumaric acid, and citric acid levels in the TCA cycle were up-regulated in NJ. Besides this, d-sucrose was significantly degraded, and the levels of d-fructose and d-glucose increased significantly after storage. GSH, l-glycine, and l-cysteine related to glutathione and l-cysteine metabolism were also quantified. GSH, which represents the reducing ability of rice, decreased significantly in both varieties after storage. l-glycine was up-regulated and l-cysteine was down-regulated significantly only in NJ. The primary oxidation products of linoleic and α-linolenic acid including 13-KODE and 9(S)-HOTrE were up-regulated in NJ.

## 4. Discussion

**Figure 7:**
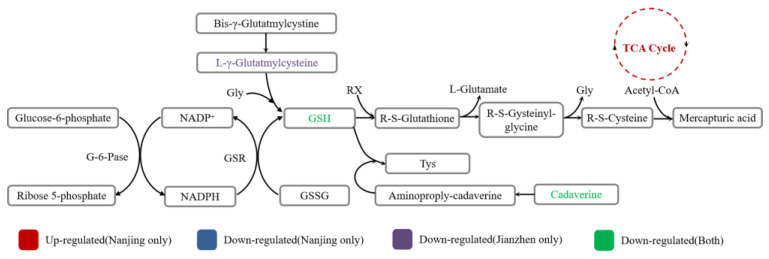
Changes in glutathione metabolic pathway of two varieties.

**Figure 8:**
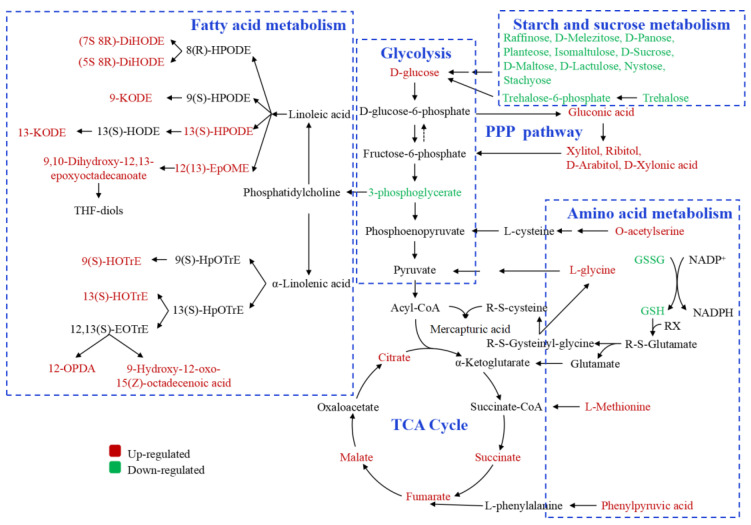
Changes in metabolites mapped to the metabolic pathways of NJ.

The current data indicate that the NJ (*japonica* rice) showed a serious deterioration in quality compared with JZ (*indica* rice). Different metabolites involved in storage can be divided into 10 classes based on their metabolic functions (Table S2). Metabolic pathways of amino acids, carbohydrates, and fatty acids are presented to compare differences in metabolic regulation of two varieties (Figure 7 and Figure 8).

### 4.1. Amino Acid Metabolism

The results of pathway enrichment revealed that the amino acid levels showed the most significant changes between the two varieties, Amino acids related to glutathione metabolism, including *N*-acetyl-l-glutamic acid, GSH, glycine (only in NJS vs. NJF), and γ-glutamylcysteine (only in JZS vs. JZF) (Figure 7). GSH plays an important role in quenching ROS and protecting the cell from oxidative damage by participating in the TCA cycle and glucose metabolism. GSH also activates a series of enzymes, such as sulfhydryl (-SH) containing coenzyme, to accelerate the metabolism of sugars, proteins, and lipids. When subjected to environmental stress, GSH maintains the rice enzyme defense system and reduces oxidative damage [8]. During plant growth, the changes in GSH content are related to the adverse effects of environmental and oxidative stress [22], while glutathione in fruit is significantly lost during storage [23]. In this study, glutathione metabolism was inhibited in both varieties (Figure 7). Similar findings were reported in many studies about the response of plants to abiotic stress [8,24,25]. l-Cysteine improved the tolerance to abiotic stress effectively [26]. The quantitative analysis showed that l-cysteine was up-regulated in JZ (*indica* rice) and down-regulated in NJ (*japonica* rice), which established the strong defensive ability of *indica* rice against abiotic stress.

### 4.2. Carbohydrates Metabolism

Carbohydrates are a large class of substances that provide energy during bioprocess, whose accumulation is closely related to the reaction against environmental stress in plants [10]. The strong metabolism of carbohydrates is evident from the up-regulation of sugars and sugar derivatives. D-Mannitol, involved in fructose and mannose metabolism, was down-regulated in both two varieties. It could be a potential scavenger of ROS in rice due to its osmotic regulation function [27]. It has been defined as a potential biomarker in high-cadmium tolerant rice [28].

Carbohydrates’ metabolism in NJ (*japonica* rice) mainly involved galactose metabolism, glycolysis, TCA cycle, starch and sucrose metabolism, and pentose phosphate (PPP) pathway in this study. Oligosaccharides including raffinose, d-melezitose, d-panose, planteose, isomaltulose, d-sucrose, d-maltose, d-trehalose, d-lactulose, nystose, and stachyose decreased significantly, while d-mannose, d-galactose, d-fructose, d-glucose, and d-glucose 6-phosphate related to glycolysis increased during storage. However, 3-phospho- d-glyceric acid decreased in NJS compared with NJF. Intermediates participated in the TCA cycle including fumaric acid, succinic acid, and citric acid. The results based on quantitative analysis showed that both were up-regulated after storage in NJ (Figure 8). The levels of nucleotides and derivatives, including 2-deoxyribose-5′-phosphate and 2-deoxyribose-1-phosphate, and sugar alcohols, consisting of ribitol, xylitol, d-arabitol, d-glucose, gluconic acid and 3-phospho-d-glyceric acid in the PPP pathway of NJS were higher than in NJF. The levels of sucrose, trehalose 6-phosphate, and d-trehalose involved in starch and sucrose metabolism were down-regulated in NJS. d-trehalose stabilizes proteins and membranes and plays an equivalent role to osmotic cells. The down-regulation of d-trehalose indicates deterioration in the regulatory function of rice [27]. The content of D-trehalose in NJS also decreased (Figure 8), which demonstrated a stronger respiratory effect during storage. Oxidative phosphorylation-related metabolites, such as fumaric acid and succinic acid, increased, while nicotinic acid adenine dinucleotide levels decreased in NJ during storage. Sucrose, glucose, and fructose are the main forms of soluble sugar in rice [11]. Sucrose, a non-reducing sugar, is the sugar with the highest level in rice kernels and accounts for about 90% of the total sugar [29]. We concluded that the soluble sugar levels of the two varieties decreased significantly after 540 days of storage (Figure 8), which is consistent with previous studies [11]. Liu et al. [10] demonstrated that the respiratory effect was stronger in *japonica* and glutinous rice during yellowing. The sugars related to carbohydrate metabolism (e.g., pentose phosphate pathway, fructose, and mannose metabolism, galactose metabolism, oxidative phosphorylation, pyruvate metabolism) accumulated in many plants during maturity [30] and under abiotic stresses such as drought and heat [31]. However, the sucrose decreased due to its degradation under drought stress [31]. These changes were not significant in JZ (*indica* rice) during storage.

### 4.3. Fatty Acid Metabolism

Fatty acids, as another integral composition in glycerophospholipids (except for glycerides), plays a significant role in maintaining the integrity of cell membranes under various degrees of stress [32,33]. These fatty acids degrade into low-molecular-weight substances such as free fatty acids, ketones, aldehydes, and acids with an unpleasant odor, resulting in a decreased eating quality of rice [12].

Our previous studies based on rice lipidomics revealed 395 and 13 non-starch lipids and starch lipids, respectively. We identified 7 classes of non-starch lipids including fatty acids (42 species), glycerolipids (102 species), glycerophospholipids (205 species), sphingolipids (78 species), sterol lipids (18 species), saccharolipids (33 species), and prenol lipids (1 specie), among which glycerophospholipids and glycerolipids were dominant according to the number of lipid molecules. Lipids bound to starch granules naturally are known as starch lipids, which are mainly composed of LPL and free FA, while only 3 classes of starch LPL including LPC (6 species), LPE (4 species), and LPG (3 species) were identified. In contrast, additional free fatty acids (58 species) belonging to starch lipids were identified in this study. After comparing the stored samples with the fresh samples, a total of 21 species of differential lipids were obtained, of which 71.4% were free fatty acids (15 species). Interestingly, the content of free fatty acids in the two rice varieties increased after storage, including 14 in NJ and 1 in JZ (Table 2). However, no differences in free fatty acids were identified in the two rice varieties. For NJ (*japonica* rice), the levels of 5S,8R-DiHODE (derived from linoleic acid), and 9-hydroxy-12-oxo-15(Z)-octadecenoic acid (derived from α-linolenic acid) increased 13.53-fold and 12.84-fold, respectively. Metabolism of ROS), such as hydroxide radical (·OH), singlet oxygen (^1^O_2_), superoxide radical anion (O_2_^−^), and hydrogen peroxide (H_2_O_2_) was decreased due to the decline or loss of the activities of antioxidants enzymes during storage (e.g., catalase (CAT), peroxidase (POD), polyphenol oxidase (PPO), phenylalanine ammonia-lyase (PAL), superoxide dismutase (SOD)), which resulted in accumulation of ROS and increased fatty acid peroxidation in organisms [34,35]. α-Linolenic acid is an abundant unsaturated fatty acid in plants. Due to the presence of skipped diene in its structure, it can be oxidized stably in plants in a non-enzymatic manner and produce specific products such as phytosteranes and phytofurans, which are valuable biomarkers associated with harmful environmental changes and quality assurance among agricultural products like olives and almonds [36]. Raval, Mahatma, Chakraborty, Bishi, Singh, Rathod, Jadav, Sanghani, Mandavia, Gajera and Golakiya [33] reported that heat stress induces the accumulation of saturated fatty acids in membrane lipids, increasing the lipid melting temperature, and preventing a heat-induced increase in membrane fluidity. Therefore, plants will maintain the stability of cell membranes and enhance their heat resistance by increasing the saturation level of fatty acids [37]. Lipidomics analysis carried out by our team with Liaoxing and Ezhong varieties has shown that linoleic acid metabolism was the most significant active metabolic pathway involved in rice lipids during storage [13]. This may suggest that abiotic stress induces changes in the fatty acid composition of stored rice. In our study, 13-KODE related to linoleic acid and 9(S)-HOTrE related to α-linolenic acid were up-regulated in NJS, which is the group associated with decreased eating quality (Figure 8). Therefore, the specific metabolites of linoleic acid and α-linolenic acid provide insight into the selection of biomarkers associated with the deterioration in the quality of NJ. For JZ (*indica* rice), no fatty acid metabolism was enriched due to the poor quantity of different metabolites. This result was consistent with our previous reports on the lipidomics profile of rice, in which *japonica* and *indica* rice exhibited different changes during storage [13,14].

### 4.4. Quality Deterioration Mechanism of Stored Rice

Significant differences in the metabolites expressed by the two comparison groups indicate that the deterioration mechanism of the two varieties may differ during storage. The metabolite data further verified this hypothesis, because NJ (*japonica* rice) exhibited more drastic changes during storage. RVA and metabolite data further validate this conclusion based on the differences in pasting properties and metabolites between stored and fresh rice. The changes in viscosity of the two varieties were similar (Table S1), which indicates consistent effects of similar conditions on starch gelatinization characteristics of NJ and JZ suggesting that the main factor affecting RVA may be the differential metabolites expressed by the two varieties (Table 2), especially sugar alcohols including d-sorbitol, d-mannitol, gluconic acid, and dulcitol, which are related to starch metabolism.

As shown in Figure 7 and Figure 8, the abiotic stress responses of different rice varieties were dynamic, and NJ (*japonica* rice) involves more complex interactions between different regulatory levels. Currently, many studies focusing on abiotic stress responses using metabolomics have been applied to plants. The oxidative imbalance caused by amino acid metabolism [8,24,25] resulted in altered fluidity and fatty acids degradation in the cell membrane due to changes in fatty acids metabolism [33,34,35], contributing to rapid deterioration in plants. Our findings are consistent with these reports. Particularly, among the differential metabolites identified in this study, the content of free FA belonging to starch lipids increased significantly, which preliminarily verified our previous hypothesis regarding the degradation of the amylose-LPL complex to amylose-FA complex in the endosperm of rice during storage, resulting in changes of lipids profile of rice [13,14]. Nonetheless, we found that not only lipid metabolic pathways, but also carbohydrates metabolism (including glycolysis, TCA cycle, starch and sucrose metabolism, and PPP pathway) of the two rice varieties differed, which may be attributed to the different subspecies. The specific changes in targeted amino acids, carbohydrates, and fatty acids of the two varieties confirmed changes in the representative metabolic pathways of NJ during storage, including the TCA cycle, linoleic acid, and α-linolenic acid.

## 5. Conclusions

In this study, the changes in metabolite in NJ (*japonica* rice) and JZ (*indica* rice) caused by storage were compared. A total of 204 differential metabolites were identified in NJ and 26 in JZ. A few sugar alcohols (d-sorbitol, d-mannitol, gluconic acid, and dulcitol) affected starch synthesis, which resulted in changes in gelatinization characteristics of both varieties. The TCA cycle, linoleic acid, and α- linolenic were unique metabolic pathways in NJ during storage. Our study provides important insights into the mechanisms of storage in different rice varieties. The identification of 11 targeted metabolites may facilitate the development of potential biomarkers to screen rice for deterioration during storage. However, the comprehensive contribution of specific secondary metabolites to the deterioration of rice quality needs to be further studied.

## Figures and Tables

**Table 1:** Texture characteristics and pasting properties of NJ and JZ (*n* = 3, α = 0.05).

Parameters	NJF	NJS	JZF	JZS
Texture characteristics				
Hardness	2.17 ± 0.09	4.18 ± 0.05 *	2.23 ± 0.04	3.18 ± 0.02 *
Stickiness	0.71 ± 0.04	0.25 ± 0.00 *	0.30 ± 0.01	0.28 ± 0.01
Balance degree	0.17 ± 0.01	0.11 ± 0.01 *	0.14 ± 0.01	0.09 ± 0.00 *
Elasticity	0.73 ± 0.00	0.68 ± 0.01 *	0.90 ± 0.00	0.88 ± 0.00 *
Pasting properties				
Peak viscosity (cP)	1924 ± 4.7	2307 ± 0.7 *	2554 ± 30.3	2716 ± 18.5 *
Trough viscosity (cP)	1116 ± 2.6	1309 ± 8.5 *	1317 ± 23.2	1487 ± 11.5 *
Breakdown (cP)	808 ± 2.1	998 ± 7.5 *	1237 ± 31.3	1230 ± 10.1
Final viscosity (cP)	1632 ± 6.8	2012 ± 8.5 *	2289 ± 26.5	2654 ± 24.0 *
Setback (cP)	516 ± 4.2	700 ± 4.9 *	972 ± 10.0	1168 ± 14.7 *
Peak Time (min)	6.0 ± 0.0	6.0 ± 0.1	5.9 ± 0.0	5.7 ± 0.0 *
Pasting temperature (°C)	68 ± 0.1	68 ± 0.1	68 ± 0.0	70 ± 0.1 *

*: means the stored group is significantly different compared with the fresh group in the same variety.

**Table 2:** Common differential metabolites of two varieties.

Class	Index	Compounds	Type	NJS vs. NJF		JZS vs. JZF		Pathway Name
				FC	VIP	FC	VIP	
Lipids	Lmhp009773	1-α-Linolenoyl-glycerol-3-*O*-glucoside	down	0.15	1.15	0.49	1.55	/
Amino acids and derivatives	pme0075	*N*-Acetyl-l-glutamic acid	up	41.95	1.20	2.16	1.60	2-Oxocarboxylic acid metabolism; Biosynthesis of amino acids; Arginine biosynthesis
	pme1086	GSH	down	0.15	1.19	0.19	1.61	Glutathione metabolism; Biosynthesis of cofactors; ABC transporters; Cysteine and methionine metabolism
Alkaloids	pme1841	Cadaverine	down	0.06	1.20	0.33	1.56	Glutathione metabolism; Tropane, piperidine, and pyridine alkaloid biosynthesis; Lysine degradation
Saccharides and alcohols	mws0214	d-Sorbitol	up	6.68	1.20	2.48	1.58	Galactose metabolism; Fructose and mannose metabolism; ABC transporters
	mws1155	d-Mannitol	up	6.51	1.19	2.20	1.55	Fructose and mannose metabolism; ABC transporters
	pme0534	Gluconic acid	up	5.01	1.20	2.69	1.59	Biosynthesis of secondary metabolites; Carbon metabolism; Pentose phosphate pathway
	pme2237	Dulcitol	up	6.70	1.20	2.48	1.54	Galactose metabolism
Organic acids	Lmbn000612	1-Pyrroline-4-hydroxy-2-carboxylic acid	up	3.71	1.09	2.02	1.56	Arginine and proline metabolism
	pme1216	2-Picolinic acid	up	3.10	1.20	2.12	1.58	Tryptophan metabolism
Nucleotides and derivatives	mws0668	Xanthosine	down	0.25	1.20	0.26	1.62	ABC transporters; Purine metabolism; Caffeine metabolism
	pmb0981	Adenosine 5′-monophosphate	down	0.37	1.17	0.29	1.56	Biosynthesis of cofactors; Purine metabolism; Zeatin biosynthesis
Tannins	pmb2928	Gallic acid-4-*O*-glucoside	up	2.90	1.10	2.17	1.35	/

## Data Availability

The data presented in this study are available in article.

## Supplementary Materials

The following are available online at https://www.mdpi.com/article/10.3390/foods11121729/s1, Figure S1: Storage temperature and relative humidity of rice; Figure S2: The images of rice samples in this study; Figure S3: The Heatmap of all samples in this study; Table S1: Complete list of the measured metabolites in two varieties title; Table S2a: Significantly different expressed metabolites in NJS vs. NJF group; Table S2b: Significantly different expressed metabolites in JZS vs. JZF group; Table S3a: Metabolic pathways of differential metabolites in NJS vs. NJF group (Top 10 impact); Table S3b: Metabolic pathways of differential metabolites in JZS vs. JZF group. Reference [38] is cited in the Supplementary Materials.
