# Supplementary material for: Metabolomic Analysis Reveals Insights into Deterioration of Rice Quality during Storage
Source: Foods. 2022 Jun 13;11(12):1729. doi: 10.3390/foods11121729 (PMC9222621; doi:10.3390/foods11121729)
Supplement: Supplementary file 1 [file foods-11-01729-s001.zip › Supplementary-S1.pdf]

Metabolomic Analysis Reveals Insights into Deterioration of Rice Quality during Storage

Supplementary material-S1

Qian Wang, Dong Zhang, Luyao Zhao, Jianlei Liu, Bo Shang, Weiqiao Yang, Xiaoliang Duan and Hui Sun *

Academy of National Food and Strategic Reserves Administration, Beijing 100037, China; wangq@ags.ac.cn (Q.W.); zd@ags.ac.cn (D.Z.); zly@ags.ac.cn (L.Z.); ljl@ags.ac.cn (J.L.); shb@ags.ac.cn (B.S.);

ywq@ags.ac.cn (W.Y.); dxl@ags.ac.cn (X.D.)

* Correspondence: sh@ags.ac.cn

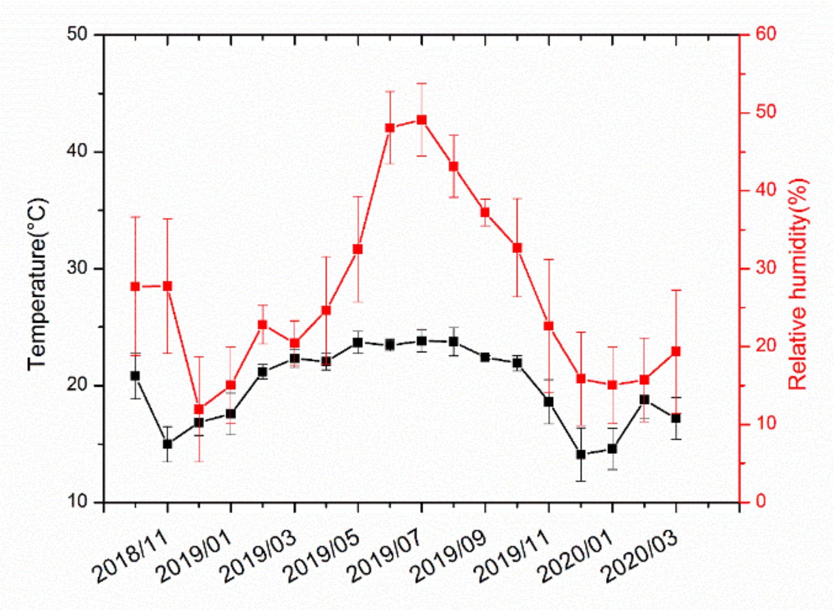

Figure S1. Storage temperature and relative humidity of rice.

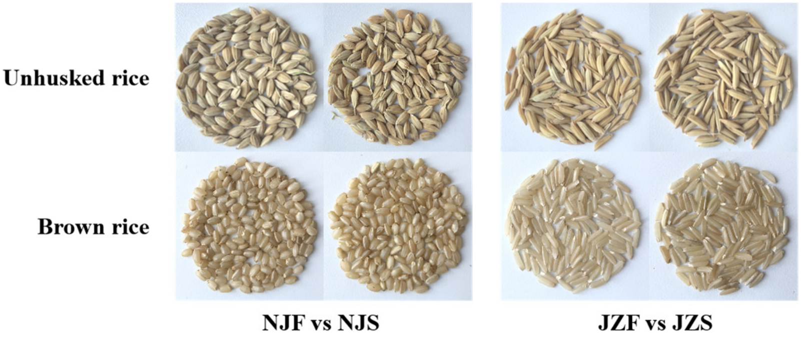

Figure S2. The images of rice samples in this study.

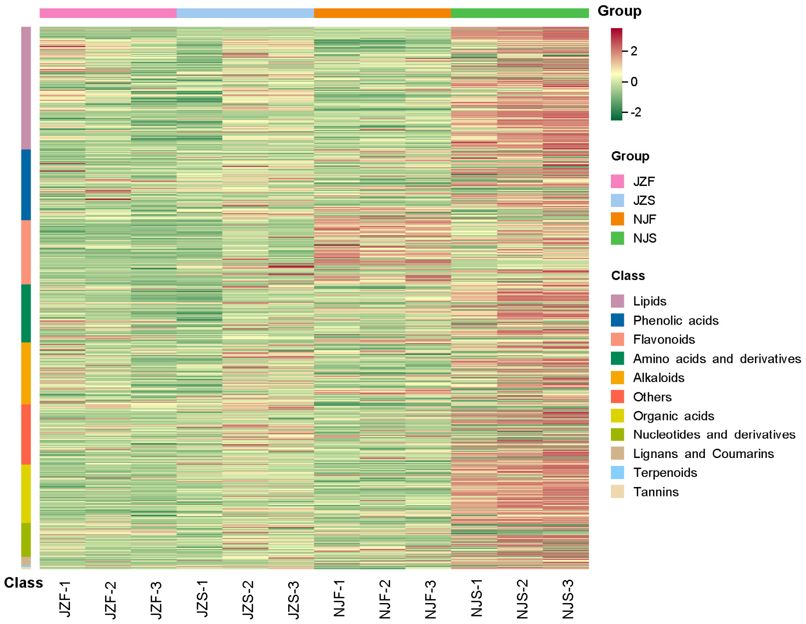

Figure S3. The Heatmap of all samples in this study.

Metabolomics data have been deposited to the EMBL-EBI MetaboLights database (DOI: 10.1093/nar/gkz1019, PMID:31691833) with the identifier MTBLS5013. The complete dataset can be accessed here https://www.ebi.ac.uk/metabolights/MTBLS5013.
