# Supplementary material for: Metabolomic Analysis Reveals Insights into Deterioration of Rice Quality during Storage
Source: Foods. 2022 Jun 13;11(12):1729. doi: 10.3390/foods11121729 (PMC9222621; doi:10.3390/foods11121729)
Supplement: Supplementary file 1 [file foods-11-01729-s001.zip › Supplementary-S2.pdf]

Metabolomic Analysis Reveals Insights into Deterioration of Rice Quality during Storage

Supplementary material-S2

Qian Wang, Dong Zhang, Luyao Zhao, Jianlei Liu, Bo Shang, Weiqiao Yang, Xiaoliang Duan and Hui Sun *

Academy of National Food and Strategic Reserves Administration, Beijing 100037, China; wangq@ags.ac.cn (Q.W.); zd@ags.ac.cn (D.Z.); zly@ags.ac.cn (L.Z.); ljl@ags.ac.cn (J.L.); shb@ags.ac.cn (B.S.); ywq@ags.ac.cn (W.Y.); dxl@ags.ac.cn (X.D.)

* Correspondence: sh@ags.ac.cn

Workflow of data analysis of rice metabolomics profile using Analyst 1.6.3 software

STEP 1: Data Processing metabolomic

UPLC-MS raw files containing full scan MS and data-dependent-MS^2 data were searched for lipids (free fatty acids, LPC, LPE, glycerol ester, sphingolipids, PC), phenolic acids, flavonoids (flavonoid, flavonoid carbonoside, flavonols, dihydroflavone, flavanols), alkaloids (alkaloids, phenolamine, plumerane, quinoline alkaloids, isoquinoline alkaloids), organic acids, amino acids and derivatives, saccharides and alcohols, nucleotides and derivatives, vitamin, lignans and coumarins (lignans, coumarins), tannins, terpenoids (terpene, triterpene, monoterpenoids) and others metabolite classes using a mass tolerance of 20 ppm for precursor ions and product ions. The search results from rice samples were aligned using a 0.25 min tolerance window and a combined report was generated.

STEP 2: Identification

For each MS^2 spectrum, search results are summarized, along with a score indicating the fit, for species matching the predicted fragmentation pattern from the database. If a mixture of metabolite is found, the most abundant metabolite is displayed. The fragment ions used to identify the metabolite species are highlighted in red when the match is selected.

STEP 3: Alignment and Quantitation

Alignment results are summarized for annotated species eluting within the

retention time tolerance window. The relative peak areas are reported along with the t-test statistics.

Instruction of DATA UPLOADING, PROCESSING, AND NORMALIZATION

for MetaboAnalyst 5.0 software

In the data filtering step, the option of "Filtering features if their RSDs are > 25% in QC samples" and "Relative standard deviation (RSD = SD/mean)" were selected. For data normalization, transformation and scaling, "Normalization by median", "Log transformation (base 10)", and "None" options were chosen and then "Normalize". Statistic continued when the characteristic "bell-shape" distribution appeared after normalization.
